# Neonatal citalopram exposure decreases serotonergic fiber density in the olfactory bulb of male but not female adult rats

**DOI:** 10.3389/fncel.2013.00067

**Published:** 2013-05-10

**Authors:** Junlin Zhang, Katie A. Dennis, Ryan D. Darling, Loai Alzghoul, Ian A. Paul, Kimberly L. Simpson, Rick C. S. Lin

**Affiliations:** ^1^Department of Neurobiology and Anatomical Sciences, The University of Mississippi Medical CenterJackson, MS, USA; ^2^Neuroscience and Cognitive Studies, Millsaps CollegeJackson, MS, USA; ^3^Program in Neuroscience, The University of Mississippi Medical CenterJackson, MS, USA; ^4^Department of Psychiatry and Human Behavior, The University of Mississippi Medical CenterJackson, MS, USA

**Keywords:** serotonin transporter, olfactory bulb, selective serotonin reuptake inhibitors, sexual dimorphism, autism spectrum disorders

## Abstract

Manipulation of serotonin (5HT) during early development has been shown to induce long-lasting morphological changes within the raphe nuclear complex and serotonergic circuitry throughout the brain. Recent studies have demonstrated altered raphe-derived 5HT transporter (SERT) immunoreactive axonal expression in several cortical target sites after brief perinatal exposure to selective 5HT reuptake inhibitors such as citalopram (CTM). Since the serotonergic raphe nuclear complex projects to the olfactory bulb (OB) and perinatal 5HT disruption has been shown to disrupt olfactory behaviors, the goal of this study was to further investigate such developmental effects in the OB of CTM exposed animals. Male and female rat pups were exposed to CTM from postnatal day 8–21. After animals reach adulthood (>90 days), OB tissue sections were processed immunohistochemically for SERT antiserum. Our data revealed that the density of the SERT immunoreactive fibers decreased ~40% in the OB of CTM exposed male rats, but not female rats. Our findings support a broad and long-lasting change throughout most of the 5HT system, including the OB, after early manipulation of 5HT. Because dysfunction of the early 5HT system has been implicated in the etiology of neurodevelopmental disorders such as autism spectrum disorders (ASDs), these new findings may offer insight into the abnormal olfactory perception often noted in patients with ASD.

## Introduction

Serotonin (5HT) is a widely distributed neuromodulator that plays an important role in regulating brain development (Gaspar et al., 2003). 5HT neurons in the dorsal raphe nucleus midline group and median raphe nucleus have axons distributed throughout the brain including the cortex (Waterhouse et al., 1986) and the olfactory bulb (OB) (McLean and Shipley, 1987). Manipulation of this system during early stages of neurodevelopment has been shown to produce sex-specific neurobehavioral modifications that persist well into adulthood (Csaba et al., 2003; Hohmann et al., 2007; Uçeyler et al., 2010). For example, serotonergic neurotoxin injections with 5,7-dihydroxytrypatmine into the bilateral medial forebrain bundles of neonatal mice induced long-term changes in social and sensory behaviors, as well as a depletion of the 5HT-immunoreactive (5HT-ir) fibers in the cerebral cortex (Boylan et al., 2007). Similarly, brief neonatal exposure to the selective serotonin reuptake inhibitor (SSRI) citalopram (CTM) lead to a sex-specific reduction of SERT-ir fibers in the neocortex (~40%) and hippocampus (~55%) of adult male rats (Maciag et al., 2006; Weaver et al., 2010). These male rats displayed reductions in novel object exploration, reduced social juvenile play (Rodriguez-Porcel et al., 2011), altered auditory sensory information processing, and reduced interhemispheric callosal connections (Simpson et al., 2011). In accordance with these findings seen in rats exposed neonatally to SSRIs, the inbred BTBR mice also display abnormalities in social juvenile play and have decreased (~20–30%) 5HT transporter (SERT) binding capacity throughout the brain (Gould et al., 2011). Depletion of 5HT in the OB affected olfactory recognition in neonate rats (Dulcy et al., 2010), and caused glomerular atrophy (Moriizumi et al., 1994). In addition, olfactory sensitivity has been shown to decrease after 3 weeks of CTM administration in adult mice (Lombion et al., 2008).

These lines of converging information demonstrate the importance of 5HT in the proper development of 5HT circuitry, and when manipulated via environmental and/or genetic means, lead to abnormal behaviors and altered innervation patterns within 5HT efferents. Therefore, our hypothesis was that brief neonatal CTM exposure will have a sex-specific effect on SERT-ir fibers within the OB, with male abnormalities being more robust.

## Materials and methods

All procedures were approved by the University of Mississippi Medical Center Institutional Animal Care and Use Committee and complied with the Association for Assessment and Accreditation of Laboratory Animal Care International and National Institutes of Health guidelines.

### Animals and tissue preparation

After the delivery of timed-pregnant Long Evans rats, the offspring were cross fostered to produce litters of 4~5 pups. The pups were tattooed for identification on postnatal day (PN) 6, weaned at PN28, and housed in groups of 2~3/cage under standard laboratory conditions with *ad-libitum* access to food and water. Beginning on PN8, the pups were injected subcutaneously with CTM (10 mg/kg, Tocris, Ellisville, MO) or saline in a volume of 0.1 ml twice daily (total CTM dose of 20 mg/kg/day or saline volume of 0.2 ml/day) for 14 days (PN8–21). This time window corresponds roughly from late stages of gestation to first 3 years of postnatal life in humans (Maciag et al., 2006). In addition, a non-treatment (NT) group (handled but without injection) was also included as a control for the effects of injection. The rationale behind the CTM dose was based on similar blood serum levels detected in rodents (Kugelberg et al., 2001) and humans (Bjerkenstedt et al., 1985), and similar dosages have been used and reported in previous rodent studies (Maciag et al., 2006; Weaver et al., 2010; Simpson et al., 2011).

After reaching adulthood (PN > 60), animals were deeply anesthetized with pentobarbital (75 mg/kg, i.p.) and perfused through the ascending aorta with 0.9% saline, followed by 3.5% paraformaldehyde in 0.1 M phosphate buffered saline (PBS). The brains were extracted and stored in 3.5% paraformaldehyde solution and 25% sucrose overnight at 4°C until slicing.

### Immunohistochemistry

Rats from each treatment group were randomly divided into subsets and then processed and analyzed in these subsets to minimize immunostaining variability. For example, three rat brains (one non-treatment, one saline exposed, and one CTM exposed) were sliced and processed on the same day. Comparison of the SERT-ir fibers in rat OB was based initially on three male experimental subsets and three female experimental subsets. Two additional female sets were added to ensure no effect of treatment since no obvious alterations were noted in the female subsets.

In general, we followed the three-step indirect immunohistochemical procedures and quantification methods described previously (Maciag et al., 2006; Weaver et al., 2010). Specifically, 60 μm coronal sections were taken throughout the OB using a freezing microtome and placed in individual wells. One out of every six sections were incubated in rabbit anti-SERT antibody (1:1000, Immunostar, Hudson, WI) for 24 h at room temperature followed by another 24 h at 4°C. After rinse, sections were incubated in biotinylated anti-rabbit IgG (1:100, BA1000, ABC kit, Vector Laboratories, Burlingame, CA) for 1 h at room temperature. Axon profiles were then visualized by incubating the sections in Cy3-conjugated streptavidin (1:200, Jackson Immunoresearch Laboratories, Inc., West Grove, PA) for 1 h at room temperature in the dark. For each step of staining within a subset, total volumes of solution were created and distributed equally across animals to eliminate variability in concentrations within subsets. Finally, sections were mounted on gelatin-coated slides, allowed to air dry, and were then covered with DPX (mixture of distyrene, plasticizer, and xylene).

To control for non-specific labeling, two basic procedures were utilized. First, we conducted experiments where sections were processed according to the protocol, except that the primary SERT antiserum was omitted. Following this procedure, no SERT immunoreactivity was detected. Another control study also yielded negative immunostaining when an inappropriate secondary antibody such as biotinylated anti-mouse IgG (1:100, BA9200, ABC kit, Vector Laboratories, Burlingame, CA) was used for linkage.

### Image acquisition and analysis

Digital photomicrographs of sections containing the area of the main OB (Paxinos and Watson, 1986, bregma at 6.7 mm) were taken with a consistent exposure time at 20× magnification using a Nikon E800 epifluorescent microscope equipped with a SenSys cool camera (Roper Scientific). The 20× magnification yielded 344 × 437 μm terminal field area. For each animal, three different sub-regions of the OB were chosen: the glomerular layer, the external plexiform layer, and the granule cell layer.

A previous study suggested that the glomerular layer in the dorsal and medial region of the OB has a higher number of SERT-ir fibers than those located in the lateral region (Gomez et al., 2005). Therefore, in order to reduce the sampling bias for the glomerular layer, we routinely took four images of the SERT-ir fibers from the middle portion of both medial and lateral regions of the OB. The same strategies of acquiring pictures were used for the external plexiform and the granule cell layers.

MetaMorph imaging software (Universal Imaging Systems) was used as described previously (Maciag et al., 2006; Weaver et al., 2010) to quantify the density of the SERT-ir fibers in the OB. Briefly, the signals on the photo were first adjusted to the same brightness. After thresholding the photo, the background was excluded and the SERT fibers were selected. A rectangular region (125 × 100 μm for the glomerular layer, 437 × 295 μ m for the external plexiform layer, and 437 × 280 μm for the granule cell layer) was created and placed on the photograph to include the specified layer. The area of this region also served as the reference point. The percentage of accumulated area of SERT fibers divided by the area of the rectangular region was defined as the SERT fiber density within this specific layer. The fiber density for each layer of an individual animal was determined by averaging the densities obtained from the four images. Data were analyzed statistically using MANOVAs with *post-hoc* Tukey's HSD tests (SPSS 19, IBM).

## Results

### Distribution and morphology of SERT-ir fibers

**Figure 1:**
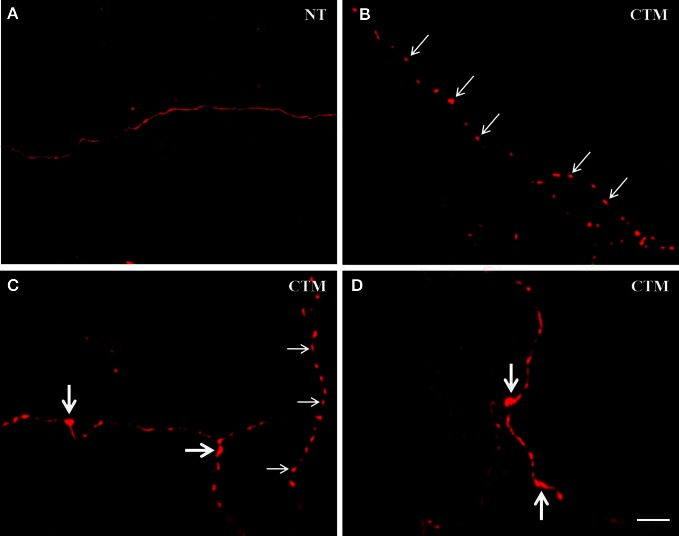
**Representative photomicrographs showing the altered morphology of SERT-ir fibers in the OB of male rats. (A)** Normal SERT-ir fibers exhibit a smooth and fine morphology with very few varicosities. **(B,C)** Beaded-like SERT-ir fibers (thin arrows) were noted in CTM exposed male rats. **(C,D)** The SERT-ir fibers with large bouton-like appearance (thick arrows) were noted in CTM exposed male rats. Scale bar = 2 μm. NT, non-treatment; CTM, citalopram; OB, olfactory bulb; SERT, serotonin transporter.

In both male and female control rats, the majority of SERT-ir fibers appear rather smooth and fine in caliber with very few varicosities (Figure 1A). Most of SERT-ir fibers in the granule cell layer tended to run parallel to the pia surface, and many of the SERT-ir fibers in the external plexiform layer were either perpendicular or parallel to the pia surface. In contrast, SERT-ir fibers in the glomerular layer were mostly randomly distributed. This general pattern of SERT-ir fiber distribution did not change in CTM exposed animals.

In contrast to the smooth and fine morphology of the SERT-ir fibers commonly seen in control animals, sex-specific varicosities were frequently noted in the glomerular layer of CTM exposed male animals (Figures 1B,C). These varicosities were found less frequently in the external plexiform and the granule cell layers. The distance between these varicosities along the SERT-ir fibers was usually very close and they were mainly in the range of ~1–3 μm apart. In addition, enlarged bouton-like swellings (Figures 1C,D) were occasionally noted in the granule cell layer of CTM exposed male rats. In contrast to these layer-specific morphological changes in male rats neonatally exposed to CTM, such changes were not seen in the female populations.

### Density of the SERT-ir fibers in male rats

**Figure 2:**
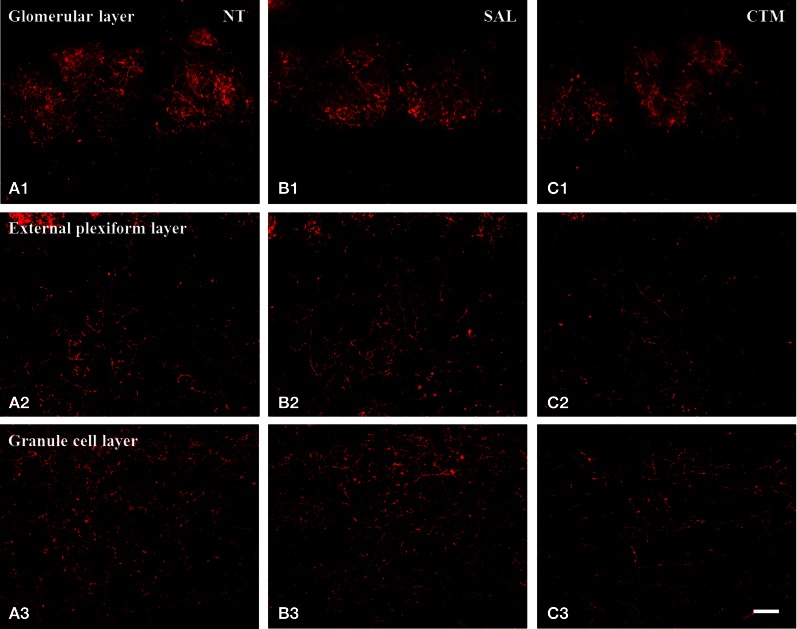
**Representative photomicrographs illustrating the distribution pattern of SERT-ir fibers in the OB of male rats. (A1–C1)** The glomerular layer. **(A2–C2)** The external plexiform layer. **(A3–C3)** The granule cell layer. Note that SERT-ir fiber density in CTM **(C1–C3)** exposed rats was reduced in all three layers compared to NT **(A1–A3)** and SAL **(B1–B3)** exposed male rats. Scale bar = 50 μm. NT, non-treatment; SAL, saline; CTM, citalopram; OB, olfactory bulb; SERT, serotonin transporter.

**Figure 3:**
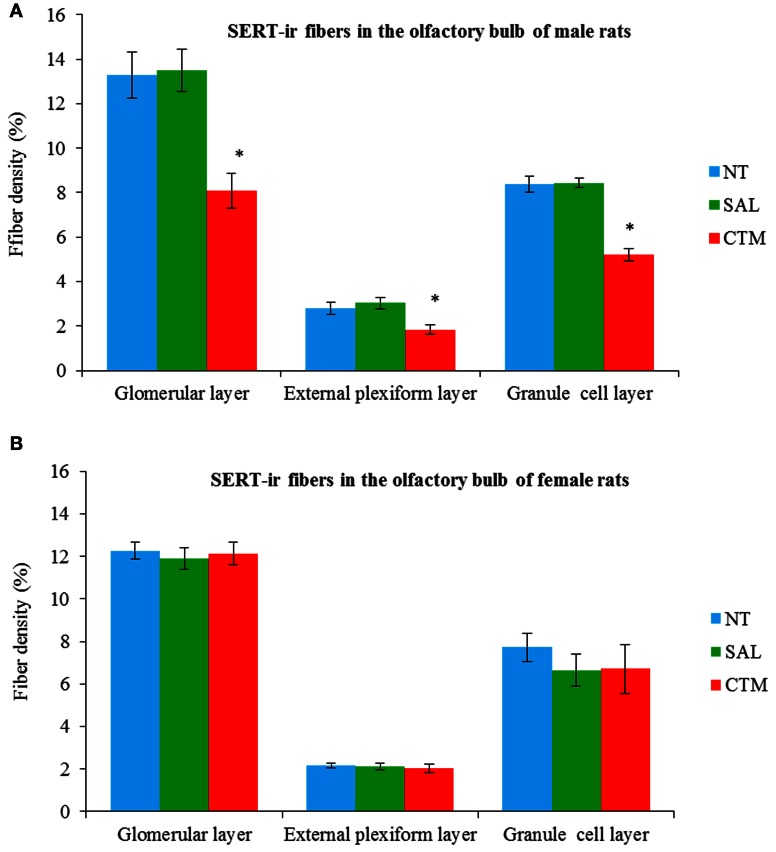
**Quantitative analysis of SERT-ir fiber density in the OB of both male and female rats. (A)** The density of the SERT-ir fibers was significantly decreased in all three layers of the OB in CTM exposed male rats compared to SAL exposed male rats. **(B)** However, such changes were not significantly different in female rats. Error bars = SEM. ^*^Represents *p* < 0.05 compared to SAL group. NT, non-treatment; SAL, saline; CTM, citalopram; OB, olfactory bulb; SERT, serotonin transporter.

Representative photos demonstrating the alteration of SERT-ir fiber density in the OB of male rats were shown in Figure 2. A one way MANOVA revealed a significant effect of treatment in the density of SERT-ir fibers within the glomerular layer of male rats, *F*_(2, 6)_= 10.82, *p* = 0.010, within the external plexiform layer, *F*_(2, 6)_ = 6.47, *p* = 0.032, and within the granule layer, *F*_(2, 6)_ = 38.81, *p* < 0.000. Compared to SAL exposed rats, Tukey's HSD *post-hoc* tests revealed that SERT-ir fiber density within the OB of CTM exposed male rats was significantly reduced ~39% in the glomerular layer (*p* = 0.015), ~38% in the external plexiform layer (*p* = 0.034), and ~38% in the granule cell layer (*p* = 0.001) (Figure 3A). Importantly, there were no significant differences in SERT-ir fiber density between NT and SAL exposed rats within any of the three OB layers (*p* ≥ 0.79) (Figure 3A), demonstrating that the effect was not due to the injections. The data from each experimental subset of male rats are listed in Table 1.

**Table 1:** **SERT-ir fiber density in the olfactory bulb of male rats**.

**Subset**	**Glomerular layer (%)**			**External plexiform layer (%)**			**Granule cell layer (%)**		
	**NT**	**SAL**	**CTM**	**NT**	**SAL**	**CTM**	**NT**	**SAL**	**CTM**
1	11.8	13.0	6.6	3.0	2.5	1.9	7.7	8.7	5.4
2	15.3	15.3	8.4	3.1	3.3	2.1	8.5	8.0	5.6
3	12.7	12.1	9.2	2.2	3.2	1.4	8.9	8.5	4.6
Mean	13.3	13.5	8.1	2.8	3.0	1.8	8.4	8.4	5.2
SEM	1.0	0.9	0.8	0.3	0.2	0.2	0.4	0.2	0.3

*NT, non-treatment; SAL, saline; CTM, citalopram; SEM, standard error of the mean*.

### Density of the SERT-ir fibers in female rats

**Figure 4:**
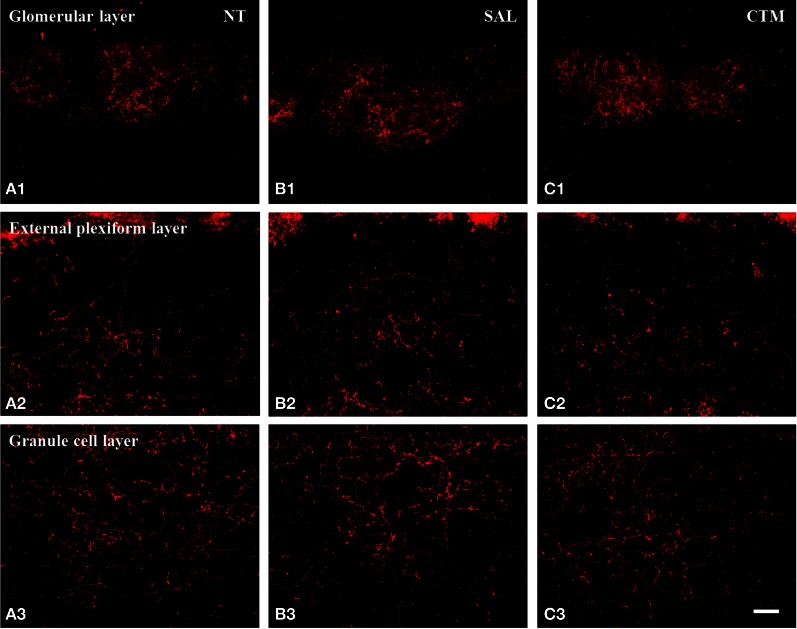
**Representative photomicrographs illustrating the distribution pattern of SERT-ir fibers in the OB of female rats. (A1–C1)** The glomerular layer. **(A2–C2)** The external plexiform layer. **(A3–C3)** The granule cell layer. Note that SERT-ir fiber density remained unchanged in all three layers of NT **(A1–A3)**, SAL **(B1–B3)**, and CTM **(C1–C3)** exposed female rats. Scale bar = 50 μm. NT, non-treatment; SAL, saline; CTM, citalopram; OB, olfactory bulb; SERT, serotonin transporter.

Interestingly, SERT-ir fiber density within the OB of female rats showed a different pattern compared to that discovered in male rats. Representative photos were shown in Figure 4. Specifically, a one way MANOVA revealed a non-significant effect of treatment within the glomerular layer *F*_(2, 12)_ = 0.173, *p* = 0.843, within the external plexiform layer *F*_(2, 12)_ = 0.300, *p* = 0.746, and within the granule layer *F*_(2, 12)_ = 0.464, *p* = 0.640 (Figure 3B). The data from each experimental subset of female rats are listed in Table 2.

**Table 2:** **SERT-ir fiber density in the olfactory bulb of female rats**.

**Subset**	**Glomerular layer (%)**			**External plexiform layer (%)**			**Granule cell layer (%)**		
	**NT**	**SAL**	**CTM**	**NT**	**SAL**	**CTM**	**NT**	**SAL**	**CTM**
1	11.9	11.8	13.6	1.9	2.6	2.6	8.8	5.3	10.4
2	12.2	12.1	11.1	2.0	2.2	1.6	8.1	7.5	7.3
3	12.1	10.0	11.2	2.2	1.8	1.6	6.1	7.3	6.2
4	11.4	13.2	11.6	2.3	2.2	2.4	9.3	8.6	6.5
5	13.8	12.3	13.2	2.5	1.7	1.8	6.3	4.6	3.1
Mean	12.3	11.9	12.1	2.2	2.1	2.0	7.7	6.7	6.7
SEM	0.4	0.5	0.5	0.1	0.2	0.2	0.7	0.7	1.2

*NT, non-treatment; SAL, saline; CTM, citalopram; SEM, standard error of the mean*.

## Discussion

We examined the neurodevelopmental effects of neonatal CTM exposure on the expression of SERT-ir fibers in the OB of adult rats. Our data revealed that disruption of the 5HT system during early life lead to a sex-specific and long-lasting change in the morphology and density of the SERT-ir fibers within the OB of adult male rats. This suggests a sexually dimorphic response to altered levels of neonatal 5HT, which is consistent with previous rodent studies (Csaba et al., 2003; Hohmann et al., 2007; Uçeyler et al., 2010), and further supports this model in the etiology of neurodevelopmental disorders (Rodriguez-Porcel et al., 2011; Simpson et al., 2011).

### Technical considerations

It has been well-documented that the 5HT immunostaining patterns within the OB are reliably represented by using SERT immunostaining, but minor differences between the two biomarkers have been noted. For example, a greater number of SERT-ir fibers have been observed in the infraglomerular layer of the OB compared to 5HT-ir fibers, and the authors suggested that 5HT immunostaining may not effectively reveal neuronal processes in cells with a low concentration of intracellular 5HT (Gomez et al., 2005). Furthermore, the SERT protein was found within serotonergic axon bundles in addition to axon terminals, which makes it a useful biomarker for examining serotonergic axons (Zhou et al., 1998).

The layer-specific density of 5HT-ir fibers in the OB of normal adult male rats has been reported to be ~8.8% in the glomerular layer, ~3.5% in the granule cell layer, and ~1.8% in the external plexiform layer (McLean and Shipley, 1987). In the current study, a similar pattern of the SERT-ir fiber density was observed in control animals with ~13.3% in the glomerular layer, ~8.4% in the granule cell layer, and ~2.8% in the external plexiform layer, supporting the relative reliability of these two immunomarkers.

Appropriate measures were employed to ensure that our analysis was not biased by our staining and sampling procedures. For example, image quantification was done independently by two investigators blind to experimental conditions in order to verify interrater reliability. In addition, the staining procedures were processed in sets to minimize staining variability across groups. In fact, the Levene's test of equality of error variances was not significant within any of the three OB layers of male rats (*p* ≥ 0.604) or female rats (*p* ≥ 0.113), demonstrating equal error variances across groups.

### Comparison of SERT-ir fiber morphological changes with other studies

In the present study, varicosities (~1–3 μm apart) on SERT-ir fibers were frequently noted in the glomerular layer of CTM exposed male rats, while these varicosities were less often found in the external plexiform and the granule cell layers. This is consistent with previous studies that found more 5HT-ir fibers with varicosities (~2–20 μm apart) in the glomerular layer compared to the infraglomerular layer (McLean and Shipley, 1987), and SERT-ir fibers with varicosities within the glomerular layer (Gomez et al., 2005) of normally developing rodents. The increased prevalence of dystrophic fibers seen in CTM exposed animals suggests altered function and may impair serotonergic signaling throughout the brain.

An increased number of dystrophic thick SERT-ir fibers has been noted in the amygdala, the hippocampus, and the cortex of autistic patients (Azmitia et al., 2011). Interestingly, rodents exposed neonatally to CTM showed an increased number of thick and rod-like SERT-ir fibers and/or fine and beaded SERT-ir fibers in the cortex and hippocampus (Maciag et al., 2006; Weaver et al., 2010). However, in the present study, very few SERT-ir fibers in the OB showed this pattern after neonatal exposure to CTM. One possible explanation for this discrepancy could be that the neurochemical composition of the OB raphe projecting system may be different from cortical raphe projecting system. For example, the majority of cortical projecting 5HT neurons are in the midline subgroup of the raphe complex co-express nitric oxide and 5HT (Simpson et al., 2003; Lu et al., 2010), and it is currently unknown whether this is the case for the OB projecting raphe neurons.

### Sexual dimorphism of the 5HT system

At present, a limited number of studies have examined sexual differences within the developing 5HT system of human or rodent brains. It was reported that cortical 5HT content in normal male mice was twice that of female mice at PN3 (Connell et al., 2004). In healthy young humans, 5HT synthesis capacity was higher in boys compared to girls (Chugani et al., 1999), and similarly higher in healthy adult men compared to women (Sakai et al., 2006). In contrast, 5HT levels were reported to be higher in the brainstem and limbic forebrain of female rats compared to male rats (Carlsson and Carlsson, 1988).

The major support for the sexually dimorphic development of the 5HT system is from rodent studies that have demonstrated sex-specific effects after early life manipulation of brain 5HT levels. For example, it was reported that 5HT depleted male animals showed reduced exploration in response to spatial rearrangement and object novelty, however, this effect was not found in females (Hohmann et al., 2007). Similarly, physical activity was reduced in adult male SERT knock-out mice compared to females (Uçeyler et al., 2010). Furthermore, after a single injection of 5HT into newborn rats, 5HT levels were reduced in the striatum of adult male rats and displayed increased sexual activity, while no obvious differences were detected in adult female rats (Csaba et al., 2003). Interestingly, perinatal exposure to CTM affected male locus coeruleus circuit function but was not seen in females (Darling et al., 2011). Because a variety of early-life 5HT-system manipulations produce a constellation of long-term and sex-specific effects, it is not surprising that we observed a sex-specific reduction of SERT-ir fiber density in the OB of only male rats after neonatal CTM exposure.

Taken together, the current investigation and previous studies suggest that a sexual dimorphism does exist in both the normally developing 5HT system and after manipulations of brain 5HT levels. At present, the precise biological mechanism(s) of such sexual differences during early development are not known. Nonetheless, the neurodevelopmental consequences of these sexual differences need to be explored as it pertains to neurodevelopmental and psychiatric disorders that exhibit sex specificity.

### Functional significance and clinical implications

Recently, our laboratory and others have suggested that manipulation of perinatal 5HT levels induce numerous neurological and behavioral abnormalities, similar to what have been observed in autistic patients. For example, patients with autism spectrum disorder (ASDs) have been known to show impaired social interactions (Wing and Gould, 1979), a disrupted 5HT system in the brain (Chugani et al., 1999; Makkonen et al., 2008; Nakamura et al., 2010), as well as under-connectivity between two cortical hemispheres (Just et al., 2004; Vidal et al., 2006; Courchesne et al., 2007; Damarla et al., 2010). In addition, a recent study reported that exposure to SSRIs during pregnancy increased the risk of ASD diagnosis by a factor of more than two (Croen et al., 2011). These new lines of evidence suggest that dysregulation of 5HT levels may be one of the contributing factors for ASDs.

In addition to the core symptoms commonly seen in ASD, autistic patients are also known to display high incidence of sensory abnormalities (Rogers and Ozonoff, 2005). For example, it was reported that over 90% of children with ASDs had multiple sensory abnormalities (Leekam et al., 2007). Abnormal responses to multisensory information such as touch, oral, and olfactory stimuli have been proposed as predictors of the social severity in children with ASDs (Hilton et al., 2010). Other studies reported either disrupted olfactory identification (Suzuki et al., 2003; Bennetto et al., 2007; May et al., 2011) or altered odor sensitivity (Tomchek and Dunn, 2007; Dudova et al., 2011) in patients with ASDs. At present, the neurochemical and neuroanatomical foundations of the altered olfactory information processing in patients with ASDs remain unclear, but this model of early-life 5HT manipulation may help elucidate the cause of these widespread sensory abnormalities.

Lastly, 5HT has been shown to play an important role in the rodent olfactory system, and disruption of 5HT levels lead to abnormal olfactory odor transmission. For example, 5HT was reported to be necessary in conditioned olfactory learning in rats (McLean et al., 1993; Moriizumi et al., 1994; Dulcy et al., 2010), mediated through 5HT_2A/2C_ receptors (McLean et al., 1996; Hardy et al., 2005). These previous findings along with our present data suggest a potential link between an altered 5HT system in the OB and the abnormal olfactory function noted in rats and patients with ASDs. However, it may be premature to suggest that an altered serotonergic system in the OB offers a possible clinical explanation for abnormal odor information process frequently noted in patients with ASDs. Therefore, additional behavioral tests of olfactory function in our animal model warrant further investigation.

### Conflict of interest statement

The authors declare that the research was conducted in the absence of any commercial or financial relationships that could be construed as a potential conflict of interest.
